# Impact of Xylose on Dynamics of Water Diffusion in Mesoporous Zeolites Measured by NMR

**DOI:** 10.3390/molecules26185518

**Published:** 2021-09-11

**Authors:** Madison L. Nelson, Joelle E. Romo, Stephanie G. Wettstein, Joseph D. Seymour

**Affiliations:** 1 Department of Physics, Montana State University, Bozeman, MT 59717, USA; madison.nelson1@montana.edu; 2 Department of Chemical and Biological Engineering, Montana State University, Bozeman, MT 59717, USA; jojoromo@gmail.com (J.E.R.); stephanie.wettstein@montana.edu (S.G.W.)

**Keywords:** NMR diffusometry, zeolites, heterogeneous catalysis sugar conversion, biomolecules

## Abstract

Zeolites are known to be effective catalysts in biomass converting processes. Understanding the mesoporous structure and dynamics within it during such reactions is important in effectively utilizing them. Nuclear magnetic resonance (NMR) *T*_2_ relaxation and diffusion measurements, using a high-power radio frequency probe, are shown to characterize the dynamics of water in mesoporous commercially made 5A zeolite beads before and after the introduction of xylose. Xylose is the starting point in the dehydration into furfural. The results indicate xylose slightly enhances rotational mobility while it decreases translational motion through altering the permeability, *K*, throughout the porous structure. The measurements show xylose inhibits pure water from relocating into larger pores within the zeolite beads where it eventually is expelled from the bead itself.

## 1. Introduction

Pulsed gradient spin echo (PGSE), or pulsed field gradient (PFG) nuclear magnetic resonance (NMR) is a preeminent method for characterization of transport and structure in porous media systems [1,2]. Despite the fact that application to nanoporous systems is challenging, due to the small structural length dimensions which generate complex rotational and translational molecular dynamics over a hierarchy of scales, significant characterization of systems such as zeolites has been attained [3,4,5,6]. The application of zeolites in catalytic conversion of biomass to fuel and chemical products is an area of growing application [7], and recent research has shown that zeolite beads have the potential to catalyze sugar to furan dehydration reactions [8]. Studies of water molecular dynamics in zeolites in the presence of biomolecules by NMR have been limited to solid state NMR spectroscopy, PGSE NMR using a single displacement observation time [9], and PGSE NMR to study water in zeolites [10,11] while solid state NMR has also been used to study solvents in zeolites [12]. Here displacement time-dependent PGSE NMR was applied to study the impact of xylose on water dynamics in zeolites for heterogeneous catalysis of sugars to furans [8,13].

Zeolites are known for their chemical and thermal stability, versatility [14], and have been widely used in biomass conversion reactions [15,16,17,18] including xylose dehydration to furfural. For example, research by Gao et al. found xylose dehydration reactions with ZSM-5 resulted in furfural yields of 51.5% in an aqueous system [17]. Other researchers have looked at the use of powdered silicoaluminophosphates (SAPOs), a class of small-pore zeolites, in various solvent systems to maximize furfural production from xylose, achieving moderate yields [15]. In order to improve catalyst recovery, Romo et al. used dual-layered zeolite beads (versus powdered zeolites) to convert xylose to furfural and achieved yields of up to 45%, indicating zeolite beads have the potential for sugar upgrading [8]. Although microporous zeolites beads are promising for sugar dehydration reactions, the zeolite pore size can create a diffusion limited system. This is particularly true for substates such as sugars, which have large kinetic diameters.

Commercially available Linde Type A (LTA) zeolite beads consist of ~3 μm crystallites made up of ~5Å molecular cages, which are compressed with binder into 3 mm beads that are 86% microporous [8]. Molecular transport in zeolite systems of this type of structure have been modeled as porous media with periodic permeable inclusions [6,19,20]. This results in an effective diffusivity 1Deff=1Do+1Kl where $D_{o}$ is the molecular diffusion within a pore structure and *K* is the permeability, reflecting transport resistance between pore structures separated by length scale *l* [19,20]. In the zeolite system studied here, $D_{o}$ is the diffusion within the zeolite crystal and *K* the permeability at the zeolite crystal grain interfaces within the 3 mm bead and $D_{eff}$ the NMR measured diffusion.

Diffusion measurements by NMR are obtained by application of magnetic field gradient pulses which attenuate the voltage signal due to magnetization dephasing caused by random diffusive motion. The measured signal normalized by the signal with no gradient is given by $E\left(g,∆\right)=\frac{S\left(g,∆\right)}{S\left(0,∆\right)}=\exp\left[-\gamma^{2}g^{2}\delta^{2}D\left(\Delta -\frac{\delta }{3}\right)\right]$, where *γ* is the gyromagnetic ratio, g is the gradient amplitude, *δ* is the gradient duration, and Δ is the gradient separation, which is the time the nuclei are allowed to displace. PGSE NMR thus measures the time-dependent effective diffusivity *D*(Δ), which characterizes the length scale of the restricted diffusion dynamics of a fluid in a pore at short times, as [2,21,22].

(1) D(∆)=Do[1−49πSV(Do∆)12]

Here *D_0_* is the free liquid diffusion, Δ the PGSE NMR displacement time for the spins, and *S*/*V* = 3/*R* the surface to volume ratio of a spherical pore of characteristic length scale radius *R*. While the normalized form of the measured signal, E(g,∆), factors out *T*_1_ spin-lattice and *T*_2_ spin–spin magnetization relaxation effects, the measured signal is weighted by the *T*_2_ relaxation if *T*_2_ times are present which are less than the PGSE echo time, as the signal from those spins are fully relaxed before being encoded for diffusive motion. *T*_2_ relaxation is due to dipolar coupling of the NMR active spins, ^1^H protons in the experiments conducted in this work, and interaction with solid surfaces in porous media. Longer *T*_2_ relaxation times occur when the dipolar coupling is averaged out by rotational diffusion, as in liquids, and shorter when rotational mobility is restricted.

## 2. Results

**Figure 1:**
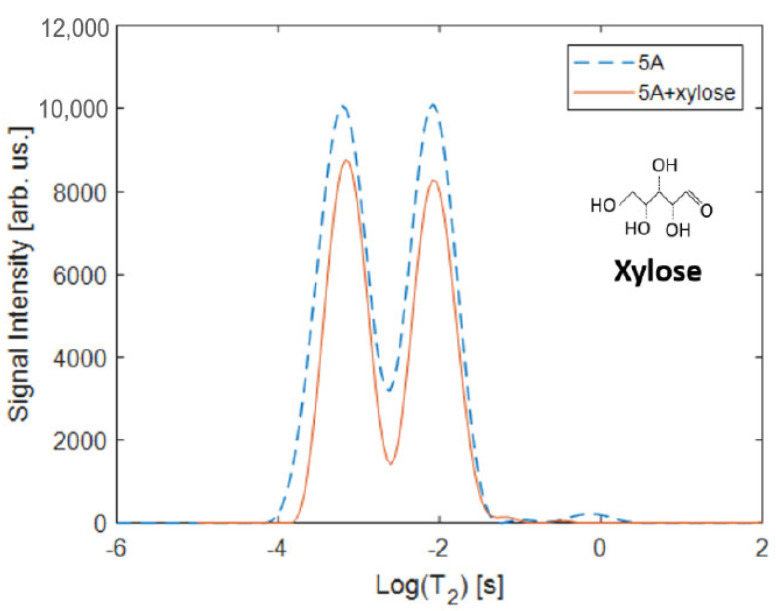
*T*_2_ distributions comparing the core zeolite Grace 5A beads before and after the addition of 20% wt. xylose to the water permeating through the system. In the 5A beads with water there are two large populations at *T*_2_ = 6.193 × 10^−4^ s and 8.300 × 10^−3^ s and a small population at longer *T*_2_ = 0.806 s. The presence of xylose eliminates the longer relaxation component and slightly shifts the two dominant populations to slightly longer relaxation times *T*_2_ = 7.518 × 10^−4^ s and 9.100 × 10^−3^ s. The 20% wt. xylose in water solution has a large relaxation population at *T*_2_ = 1.167 s and a small population at 1.260 × 10^−1^ s representing the biopolymer and water relaxation times (not shown). The chemical structure of xylose is shown in the upper right.

*T*_2_ relaxation measurement of water xylose solution in bulk has a large peak at 1167.1 ms due to water and a small peak at 126 ms from the xylose. *T*_2_ relaxation of pure water and of the 20% wt. xylose solution in the 5A bead indicate two primary populations of relaxation behavior as shown in Figure 1. The fast relaxing, short *T*_2_ relaxation populations are at 0.619 ms for water and 0.752 ms for the xylose solution. These sub millisecond relaxation times demonstrate the significant restriction of rotational mobility and interactions of primarily water and the zeolite surfaces (kinetic diameter 2.7 Å) [23] in the zeolite micropores, since the xylose (kinetic diameter 6.8 Å) [24] is too large to be within those pores. The presence of the xylose generates an increase in the *T*_2_ relaxation time. The slower relaxing, longer *T*_2_ populations in the zeolite beads at 8.300 ms for pure water and 9.100 ms for the xylose solution are due to the molecules in the inter-crystalline mesopores of the bead. In this more mobile population, the presence of the xylose induces a slight increase in the rotational mobility of the protons in the system. In the pure water in zeolite system there is a small peak at 806 ms which is associated with water in macro pores or leaking to the bead surface, while this population is suppressed in the xylose solution system. Due to only 20% of the solution being xylose, the signal is primarily from water. Spectral resolution is not possible in the zeolite beads due to the signal broadening caused by the restrictions of the solution within the zeolite, and the *T*_2_ relaxation distributions show little signal changes after the introduction of xylose. Therefore, the NMR signal obtained will be primarily attributed to water within the system.

**Figure 2:**
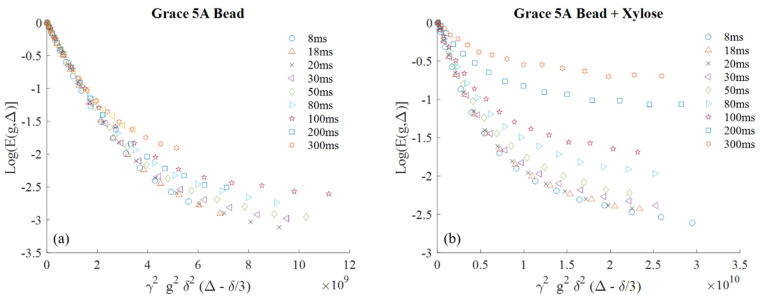
Stejskal–Tanner plots of Grace 5A beads (**a**) and Grace 5A beads with xylose (**b**) are shown. With increasing displacement observation time Δ from 8 to 300 ms, the attenuation of signal decreases for the pure water and xylose solution saturated beads. The gradient duration δ was 1 ms with a maximum gradient ranging from 0.500 to 1.9021 T/m in order to sample out to a similar point in the gradient domain. The data are well fit by a biexponential model with a fast and slow diffusion component $p_{f}exp\left[-D_{f}x\right]+p_{s}exp\left[-D_{s}x\right]$.

The displacement time dependent pulse gradient stimulated echo (PGStE) NMR signal attenuation data as a function of increasing pulsed gradient strength is shown in Figure 2. The data are plotted in a standard Stejskal–Tanner plot format in which the slope of the curve indicates the diffusion coefficient [2]. The data exhibits biexponential behavior with a fast and slow diffusing component. It is important to note that the ^1^H proton signal measured comes only from the longer *T*_2_ relaxation population since the sub millisecond relaxing populations are filtered out by the 4.32 ms echo time of the stimulated echo experiment. The slow diffusing component increases in quantity as a percentage of the total signal as the displacement observation time is increased.

**Figure 3:**
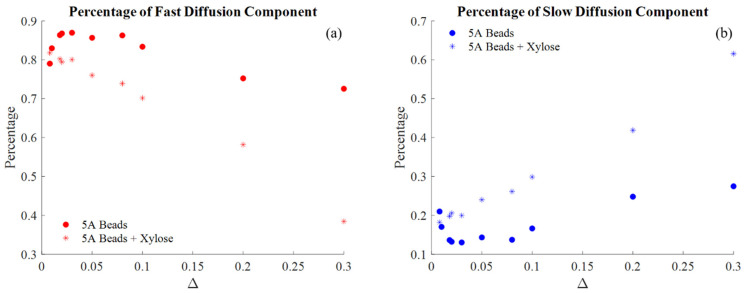
The population percentage of the fast (**a**) and slow (**b**) diffusion populations with varying Δ. The signal is weighted toward the slower diffusing regions before and after the addition of the xylose with increasing Δ.

This can be seen by fitting a biexponential model $p_{f}exp\left[-D_{f}x\right]+p_{s}exp\left[-D_{s}x\right]$ to the data and determining the population in the fast and slow decay regions, Figure 3. In the pure water system, there is an initial increase in the amount of fast diffusion component, and commensurate decrease in the slow diffusion population, which is associated with the water moving into larger pore regions of the beads during the 20 min of each initial short displacement time Δ experiment. After the Δ = 50 ms experiment, the signal proportion in the slow component increases. This can be attributed to a transient redistribution of the pure water into larger pore spaces followed by a loss of the water signal in the large pores due to dephasing of the signal or drainage from the large pores out of the sensitive region of the rf coil over the hours long experimental time for all the displacement times Δ. While any long-time scale transient redistribution of the water prohibits determining exchange between the fast and slow diffusion populations it does not negatively impact the assessment of the length scales associated with the diffusion dynamics. Of interest is the impact of the xylose in solution on the distribution of ^1^H proton signal in the fast and slow diffusion populations, in that it generates a more significant loss of fast diffusion signal. The xylose maintains more liquid within the slow diffusion population than the pure water and inhibits the initial redistribution of the water into the larger pores during displacement times Δ < 50 ms. experiments.

**Figure 4:**
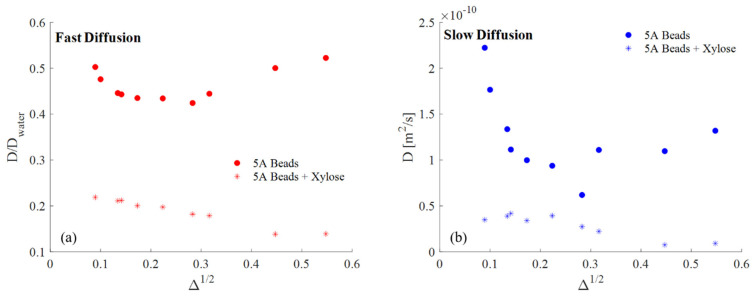
Diffusion coefficients calculated from the biexponential fits to the Stejskal–Tanner plots at each Δ, plotted against Δ^1/2^ for the zeolite beads with the pure water and xylose solution for the fast (**a**) and slow (**b**) diffusion component. The fast diffusion is plotted normalized by the free water diffusion coefficient 2.0 × 10^−9^ m^2^/s. The presence of the xylose significantly decreases both the fast and slow diffusion coefficients in the beads. The increase in diffusion of pure water in the zeolite at longer Δ indicates some nonstationary water redistribution.

The primary results of the PGStE measurement are the *D*(Δ) data for the fast and slow diffusion populations shown in Figure 4. The data are plotted against Δ^1/2^ so that the short displacement time data provide *S/V* and the long displacement time data provide the tortuosity [21,22]. The beads with pure water show an increase in diffusion coefficient for both the fast and slow component for displacement times Δ > 80 ms. This is consistent with a possible draining of the beads over the total experimental run and the loss of the fast diffusion population and precludes determination of the tortuosity, however further studies are required to determine the origin of this effect. The decrease in the fast diffusion coefficient with pure water to a value less than 1/2 that of free water indicates a diffusion length scale of the order of *l* = (2*D*Δ)^1/2^ = 9.49 μm at Δ = 50 ms, representative of multiple mesopore transport. The xylose solution is much more restricted in the largest pores and has a reduction in diffusion to less than 1/5 the free water value. The slow diffusion data for the pure water in the bead displays the classic Δ^1/2^ decay predicted by theory at short times [21,22]. Determination of the length scale from Equation (1) gives R ~3.87 μm consistent with the inter-crystal mesopore length. The reduction of the slow diffusion coefficients in the xylose solution beads relative to the beads in pure water indicates a decrease in the permeability *K* at the zeolite crystal grain surfaces.

## 3. Discussion

The NMR data presented indicate the presence of a biomolecule such as xylose alters the rotational mobility of water in zeolite crystal beads slightly, while generating larger changes in translation mobility. The xylose solution alters the interplay of fluid distribution between fast and slow diffusion populations. The increase in the percentage of fluid undergoing slow diffusion in the xylose solution relative to the pure water implies that the fluid in the largest pores is more prone to drain from the beads. Any seepage from the larger pores means caution must be used in interpreting changes in the fast diffusion coefficient with displacement observation time Δ. The slow diffusion component is associated with the zeolite crystal length scale. The slow diffusion coefficients measured for the pure water and the xylose solution indicate the xylose decreases the permeability at the zeolite crystal interfaces. The xylose also makes the slow diffusion behavior less time dependent over displacement timescales from Δ = 8 to 100 ms than in pure water, consistent with a decrease in permeability restricting water translational diffusion even on shorter timescales. This demonstrates that xylose dehydration reactions in zeolite bead catalysts can be impacted by diffusion limitations, even with mesoporosity.

## 4. Materials and Methods

Commercially obtained Zeolite 5A beads (W. R. Grace & Co., Columbia, MD, USA) are composed of 4.2 Å molecular cages grown to ~3 μm crystallites indicated by manufacturer’s data and then compressed with binder into 3 mm beads. The exact crystal size within the bead is not measured, but there is crystallinity as seen by the XRD reported in our previous manuscript [8]. The beads were first saturated with water by placing 0.3 g of beads into a 15 mL pressure tube. Approximately 4 g water and a stir bar were added to the tube and then sealed with a Teflon cap with front-seal Kalrez o-ring. The pressure tube was then placed in an oil bath set at 443 K for 10 min with a stir rate of 600 rpm. The beads were then filtered from the liquid, gently blotted with a paper towel, and then placed in the NMR tube. For the beads containing xylose, a similar method was used, but the water was replaced with a 20% wt. xylose solution.

After the heat treatment period the catalytic beads were placed in a 5 mm glass NMR tube were returned to room temperature and loaded into the NMR probe in the magnet. Diffusion and *T*_2_ relaxation experiments were performed in a Bruker 250 MHz superconducting magnet using a custom built high-power ^1^H rf probe and 5 mm rf coil (Bruker, Karlsruhe). Sample temperature was controlled through the Bruker BTU system with N_2_ gas flow and kept at 292 K throughout all experiments. A standard Carr–Purcell–Meiboom–Gill (CPMG) pulse sequence [1,2] was used for *T*_2_ measurements with an echo time of τ_E_ = 192 µs, 10,000 echoes, a dwell time of 4 µs, and 7.4 µs 180° rf pulses at power of 100 W. Each measurement had 64 averages. The diffusion measurements were acquired using a pulsed gradient stimulated echo (PGStE) pulse sequence [1,2] with δ = 1 ms, with maximum gradients ranging from 1.9021 T/m to 0.5003 T/m dependent on the displacement time Δ value which spanned 8–300 ms. The stimulated echo pulse sequence with an echo time of τ_E_ = 4.32 ms was averaged 64 times.

The *T*_2_ relaxation experimental data was processed through a Fredholm integral, also commonly described as an inverse Laplace transform method. The diffusion data were not analyzed through this method due to having only 16 echoes. This analysis technique works optimally with more echoes. The diffusion data were fit directly from the Stejskal–Tanner plots using a bi-exponential fitting process to obtain the diffusion coefficients.

## 5. Conclusions

NMR can provide data on the impact of biomolecules on the translational and rotational dynamics of water in zeolite beads. The significant decrease in diffusion due to decreased permeability at the zeolite crystal interfaces could impact the reaction dynamics and the catalyst performance.

## Data Availability

The data presented in this study are available within the article.

## Sample Availability

Samples of the compounds are commercially available.
